# The Importance of Mammalogy, Infectious Disease Research, and Biosafety in the Field

**DOI:** 10.13014/K27P8W9Z

**Published:** 2016-08-31

**Authors:** Matthew R. Mauldin, Jeffrey B. Doty, Yoshinori Nakazawa, Ginny L. Emerson, Darin S. Carroll

**Affiliations:** 1 Poxvirus and Rabies Branch, Centers for Disease Control and Prevention, 1600 Clifton Road NE, Atlanta, Georgia, USA; 2 Oak Ridge Institute for Science and Education (ORISE), CDC Fellowship Program, Oak Ridge, Tennessee, USA; 3 Environmental, Safety, and Health Compliance Office, Centers for Disease Control and Prevention, 1600 Clifton Road NE, Atlanta, Georgia, USA

**Keywords:** mammalogy, personal protective equipment, precautions, risks, safety, zoonoses

## Abstract

Large amounts of data and multitudes of publications have been independently generated by researchers in mammalogy and infectious diseases. The frequent confluence of these fields in epidemiological research as well as the facility of the data generated to be used in applied methods (e.g., conservation, public outreach, public health interventions) suggests that the intersection of these fields is important not only to their committed scientists but also to other areas of investigation, including public health. Given the increased frequency with which researchers in these fields interact with potentially infected humans, animals, and tissues, their occupations present a higher risk of exposure to a variety of pathogens than those in other fields of biology or among most jobs of the general public. However, a variety of methods are available for minimizing this risk, including increasing awareness of potential risks, using medical prophylaxes (when available), properly employing personal protective equipment, and using adequate disinfectants. Although instances of serious illness from zoonotic diseases among field researchers may be uncommon, they do occur; the purpose of this document is to increase awareness of risks that researchers—principal investigators and students alike—face and highlight steps and resources that can mitigate those risks.

## Introduction

Mammalogy is a broad field of study that includes such diverse disciplines as studying morphological or physiological variability (Gardner et al., 2014), understanding animal behavior (Davis et al., 1999), examining levels and patterns of genetic variability (Olayemi et al., 2012; Bradley and Mauldin, 2016), and monitoring overall trends in mammalian biodiversity (Willig et al., 2003). These disciplines provide the scientific community with a better understanding of the ecology and evolution of mammals, further improving our understanding of biology for all taxa. Previous studies have also shown that basic research often can have economic importance (Salter and Martin, 2001). To mammalogists, the increased knowledge of the natural world as well as potential economic benefits of research adequately illustrate the importance of mammalogy; however, the increased understanding of mammalian evolution and biodiversity can also influence various other fields of study, including public education (Gore et al., 2006) and wildlife conservation (Young, 1994).

Multiple studies have illustrated the high diversity of mammalian species present in the lower latitudes (Kaufman, 1995; Willig et al., 2003; Ceballos and Ehrlich, 2006). A recent publication ranked the top 19 countries with the greatest known diversity of mammalian species, and each one contains at least some landmass in the tropics (Ceballos, 2014). Given these data, it should not be surprising that the tropics are a hotbed of biological research. Additionally, this is an area of constant discovery, and the rate at which new mammal species are being identified has increased since the 1960s; furthermore, a greater number of recently identified mammalian species have been reported from the tropics than temperate regions (Reeder et al., 2006). In the same period of time, many areas of high biodiversity and endemism (in the tropics and elsewhere) have lost increasingly large portions of primary habitat because of anthropogenic activity, endangering known endemic species as well as those still unknown to science (Ceballos and Ehrlich, 2006; Myers et al., 2000). This decline highlights the need and importance for continued research of mammalian biodiversity, public education, and conservation in the tropics as well as in other parts of the world.

Researchers working with emerging infectious disease (EID) have reported similar geographical (the lower latitudes) and temporal trends (increased rate of discovery) (Jones et al., 2008). Studies have indicated that the frequency of EID events has increased since the 1940s, suggesting the threat of EIDs to global health is increasing (Jones et al., 2008; Brooks et al., 2014). Furthermore, it has been estimated that approximately 60% of EIDs are zoonotic in nature (Jones et al., 2008), and that zoonotic diseases, both emerging and established, cause an estimated 2.5 billion human illnesses and 2.7 million deaths each year (Grace et al., 2012). Given the global distribution of zoonoses along with their substantial human health implications, zoonotic disease research is an extremely active field with a wide variety of disciplines. Some research efforts have utilized algorithms to model the ecological niche of a disease (Peterson 2006) or the spread of infection (Meyers, 2007). Researchers often use genetic data to examine phylogeographic and evolutionary patterns of pathogens (Nakazawa et al., 2015); others conduct vaccine efficacy testing (Agnandji et al., 2016; Keckler et al., 2011) or examine cellular mechanisms used by antivirals (Hemmi et al., 2002).

Serological surveys have helped identify both potential reservoir/host species of zoonoses and prevalence of infection in sylvatic or urban systems (Field et al., 2001; Pitts et al., 2013; Nolen et al., 2015). When a potential mammalian reservoir is identified based on serological surveys or other investigations, mammalogists may know a great deal about the host, but a paucity of information regarding its taxonomy and/or phylogeographic patterns could be just as likely. One case of the former was the identification of *Peromyscus maniculatus* (deer mouse) as the primary reservoir of the hantavirus *Sin Nombre virus* (SNV) (Nichol et al., 1993; Childs et al., 1994). The genus *Peromyscus* is one of the most actively studied genera of North America, and this research provided information regarding the geographic distribution and ecology of the SNV reservoir, which was used to better determine additional regions and habitats that might harbor both the reservoir and virus. An example of the latter is the identification of *Cricetomys* spp. as a potential reservoir for *Monkeypox virus* (MPXV) through serological surveys as well as its involvement in the 2003 outbreak in the United States (Hutin et al., 2001; Hutson et al., 2007; Reynolds et al., 2010). Although the geographic distribution of the genus as a whole is fairly well understood, there is uncertainty in the number of recognized species. Four species are recognized by Musser and Carleton (2005), yet the most recent publication of the IUCN red list (van der Straeten et al., 2008) recognized only two species. All studies available for the listed citations relied upon morphological and ecological data. The most extensive molecular examination of the genus published to date recognized three additional lineages that likely represent previously unrecognized species (Olayemi et al., 2012), and ongoing research has identified an additional two lineages, suggesting the number of species is as high as eight (Mauldin et al., unpublished data). Those genetic analyses indicate the presence of an eastern and western clade of *Cricetomys*, similar to the geographic pattern seen in MPXV (West African and Congo Basin clades; Likos et al., 2005; Nakazawa et al., 2015), although previous understanding suggested the distributions of *Cricetomys gambianus* and *Cricetomys emini* stretched from west to east Africa. Identification of geographic barriers to gene flow of mammalian reservoirs has the potential to provide insights into geographic structure of the virus as well. This is especially interesting within the MPXV system, given the difference in virulence between the West African and Congo Basin clades of MPXV.

Through both mammalian and epidemiological studies, vast amounts of data have been generated and are frequently used to guide public health interventions to save lives and improve the quality of life for people around the globe. After the discovery of SNV in the United States, research conducted by mammalogists and epidemiologists led to a better understanding of the genetic and geographic variability of SNV and the host relationships of the species of New World hantaviruses (Nichol et al., 1993; Childs et al., 1994; Fulhorst et al., 2007; Pitts et al., 2013; Montoya-Ruiz et al., 2014). These studies prompted discussion and changes in safety regulations that affected many researchers (Fulhorst et al., 2007; Mills et al., 1995a; Mills et al., 1995b; Kelt et al., 2007). Similar research has illuminated biogeographic patterns of pathogenic South American arenaviruses (Griffiths et al., 1992; Fulhorst et al., 1997; Delgado et al., 2008; Irwin et al., 2012).

Historically, understanding routes of infection and how to minimize the risk of human exposure through large-scale vaccination campaigns led to the elimination of canine rabies from the United States. This, along with the implementation of oral rabies vaccine for geographic containment of host-associated rabies strains and the development of post-exposure prophylaxis (PEP) has decreased the number of annual human rabies deaths in the United States from approximately 100 in the early 1900s to about 2 today (Finnegan et al., 2002). Globally, an estimated 15 million people receive rabies PEP annually, which is thought to save hundreds of thousands of lives each year (WHO, 2005). These life-saving applications would not have been possible without epidemiological research to understand the natural history and modes of transmission for hantaviruses, or the host-specific and geographically distinct patterns of *Rabies virus* variants throughout North America. These data were then utilized for public outreach and the design of oral rabies vaccine campaigns.

Similar success stories are many, regarding both zoonotic and human-specific pathogens, and countless lives have been saved throughout the world. Unfortunately, there are still many diseases, specifically in the tropics (commonly referred to as “neglected tropical diseases”), which affect billions of people, primarily in developing countries (Feasey et al., 2009). Jones et al. (2008) determined that the risk of wildlife, zoonotic, and vector-borne EIDs originating at the lower latitudes is substantial and suggested better allocation of global resources to improve EID research in the tropics. As zoonoses are capable of infecting both humans and wildlife, the finding that mammalian biodiversity is a strong predictor of disease co-occurrence (Hoberg and Brooks, 2015; Murray et al., 2015) is not surprising. This correlation and the trend of increased mammalian biodiversity in the lower latitudes further supports the need for research in both mammalian diversity and zoonotic diseases in the tropics.

## Few Things Worth Doing Are without Risk

Clearly, as discussed earlier, there are many benefits to both mammalogical research and zoonotic disease research, including the generation of valuable information regarding evolution, speciation, and the ecology of their respective taxa. Additionally, these data have been used in applied fields such as conservation and mitigating risk of zoonotic diseases, among countless other implementations. However, researchers (e.g., wildlife biologists, pathologists, epidemiologists, and public health officials) and others working with potentially infected people, animals, and materials, are at a higher risk of contracting zoonotic diseases than is the general population (Zeitz et al., 1995; Mann et al., 1984; Baker and Gray, 2009). Mammalogists may not intentionally seek out infected animals, but many work in potential hotspots for EIDs or with mammals known to harbor various pathogens, placing them at higher risk of exposure to certain zoonoses.

Researchers have used serosurvey results and questionnaire responses from a variety of professional meetings (e.g., American Society of Mammalogists, Wildlife Disease Association, Southwestern Association of Naturalists) to argue both for (Fulhorst et al., 2007) and against (Kelt et al., 2007) the need for additional personal protective equipment (PPE) in the field determined by assessing the risk of occupational exposure to specific pathogens. Although the proportion of researchers exhibiting evidence of previous exposure to disease-causing pathogens may be low, there is a very real risk in working with pathogens or pathogen-associated organisms (mammals and vectors). There are multiple instances of biologists working in the field (either handling animals directly, entering areas inhabited by mammals, or simply exploring nature) becoming ill and even dying as a result of exposure to zoonotic diseases. Although some researchers in the following examples were investigating pathogens, others were not.

In 2004 a wildlife sciences graduate student working in West Virginia was infected with the *Monongahela virus* (a species of hantavirus), developed symptoms of HPS, and died shortly after hospitalization (Sinclaire et al., 2007). Lack of PPE when handling rodents and a failure to wash hands before eating were referenced as two potential exposures to the virus. The same study reported a retrospectively diagnosed 1981 case of HPS in a wildlife biologist who worked in one of the same counties (Sinclaire et al., 2007). Also in 2004, a field technician became ill with HPS in California, although it was undetermined whether the exposure occurred from direct handling of small mammals or from sleeping in a bunkhouse with rodents that tested positive for antibodies reactive to SNV (Kelt et al., 2007). One year later, two field workers became ill and were hospitalized in Boulder, Colorado, after collecting rodents for ecological studies at separate field localities (Torres-Pérez et al., 2010). A wildlife biologist died from the plague in 2007 in Arizona after conducting a necropsy on a mountain lion without the use of proper PPE (Wong et al., 2009). In 2008 two American scientists contracted *Zika virus* while working in Senegal, and person-to-person transmission was proposed when the wife of one scientist also became ill (Foy et al., 2011). In 2011, 13 biology students developed an acute respiratory illness (some were hospitalized). All are suspected to have acquired pulmonary histoplasmosis from entering a hollow, bat-infested tree in Uganda during a class field trip. Serological data confirmed diagnosis for five of the students (Cottle et al., 2013).

These are examples of infection from recognized pathogens known to occur in those areas; however, with the increased rate of EIDs, more and more diseases are being discovered (Brooks et al., 2014). One such instance was the discovery of *Sosuga virus* when a young biologist became infected with a novel paramyxovirus after fieldwork in South Sudan and Uganda. This biologist was hospitalized for two weeks with severe acute febrile disease and had lingering symptoms for months (Albariño et al., 2014; Amman et al., 2015). The exact time and location of exposure is unknown; however, it appears inconsistent adherence to use of proper PPE may have occurred. Vector-borne diseases are also a health concern, as one wildlife biologist is reported to have contracted Lyme disease three times (Moyer 2015). Collectively, these incidents occurred over wide latitudinal and elevational gradients, indicating the need for biosafety and risk mitigation in the field regardless of where fieldwork is conducted.

The point of discussing examples of field biologists becoming ill from a variety of pathogens is not an attempt to scare and manipulate researchers into unnecessarily increasing their use of PPE at the cost of comfort and mobility. The authors acknowledge that these instances are rare considering the number of researchers working in the field around the world, and many mammalogists as well as wildlife biologists have admitted to rarely (if ever) using gloves, respiratory protection, or splash guards (Fulhorst et al., 2007; Kelt et al., 2007; Bosch et al., 2013) and have no detected history of zoonotic disease infection. Rather, the reason is to remind principal investigators, as well as those entering the realm of field biology, that there are inherent risks associated with handling small mammals and to initiate a conversation regarding the most appropriate and efficient ways to mitigate those risks. It is the hope of the authors that this discussion will help researchers make informed decisions regarding the health of themselves, their colleagues, and students.

## PPE and Other Ways to Mitigate Risk of Exposure

It has been suggested that “the most important prophylactic measure for personnel who are trapping, handling, bleeding, or dissecting rodents is to be mindful of potential routes of infection and carefully avoid conditions which may lead to transmission” (Mills et al., 1995a; CDC, 1993). This practice begins with being aware of dangers and informing all participants of potential risks and methods that can minimize those risks (Mills et al., 1995a). Risk mitigation should commence well before actual fieldwork and include educating all participants of potential zoonoses or infectious diseases known to occur in the area (see CDC Yellow Book; CDC, 2016), symptoms of those diseases, and ways to avoid becoming ill (including suggested medical prophylaxes, if available) as well as proper training regarding the use of PPE, animal handling, and necropsy techniques. Discussion should also include that active handling is not the only method of potential exposure and that safety should be considered first in all field activities, including the avoidance of housing that shows evidence of occupation by rodents (Kelt et al., 2010) and not entering enclosed, poorly ventilated areas (i.e., abandoned buildings, caves, attics, hollow trees, etc.) without appropriate respiratory protection because even short exposure times in enclosed areas can result in infection with some zoonotic diseases (Tsai, 1987; Cottle et al., 2013). Wildlife biologists might be expected to know these things; however, a survey of biologists and wildlife workers revealed that many respondents admitted to not using gloves when handling potentially infectious carcasses, and 62% of respondents claimed to have no formal education on zoonotic diseases (Bosch et al., 2013).

Once all parties have been informed of the potential health risks involved in a certain type of fieldwork, training in proper techniques regarding live animal handling, non-invasive sampling, and/or necropsies can further minimize risk (Mills et al., 1995a). Proper understanding of techniques is necessary for the ethical treatment of animals (Sikes et al., 2011; Sikes et al., 2016) as well as the safety of human participants (Mills et al., 1995a). Additionally, thorough use of appropriate disinfectants is one of the most efficient means of preventing the spread of various zoonoses (Mills et al., 1995a). Use of disinfectants on traps has been recommended (Mills et al., 1995a), and despite common concern about small mammals avoiding traps cleaned with chemicals, a recent study (Wilson and Mabry, 2010) found no significant difference in trapping success between treated and untreated traps. Each of these practices (education of risks, medical prophylaxes, familiarity, and the use of appropriate techniques) provides a “layer” of protection to further reduce the risk of exposures.

**Figure 1:**
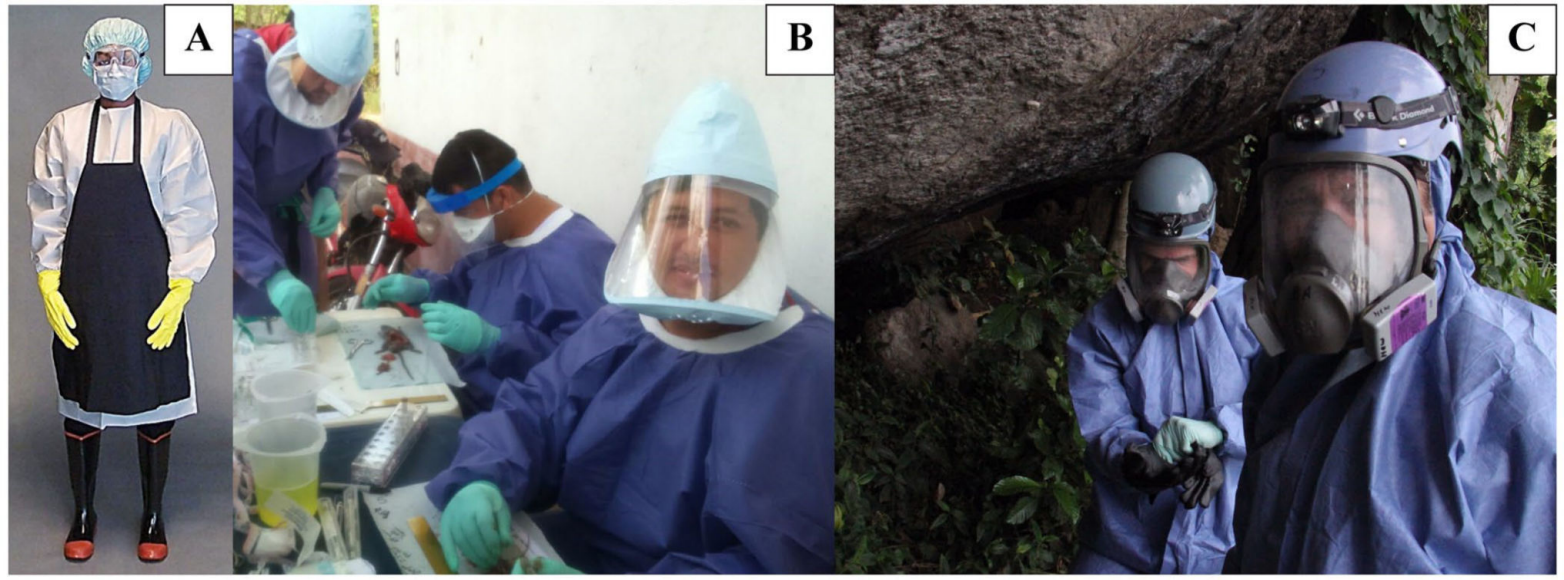
Use of PPE depends upon the situation. The PPE of an Ebola medical responder* (A) is drastically different than that needed by field researchers conducting necropsies in Colombia (B) or entering caves in Africa (C). 1C photo credit Brian Bird (CDC). *Indicates acceptable PPE for Ebola medical responders as recommended by WHO.

The proper use of PPE is yet another level of protection. The optimal amount and type of PPE will vary greatly depending upon the type of activity that is planned (Figure 1). For example, a disposable outer layer of clothes (surgical gowns, lab coats, etc.) tucked into your gloves can help reduce the risk of exposure to ectoparasites (Mills et al., 1995a). When entering or cleaning an enclosed area with evidence of small mammal activity or during active handling/necropsy conditions where particles could be aerosolized, the use of respiratory protection is advised (Mills et al., 1995a; Kelt et al., 2010). Either a powered air purifying respirator (PAPR) equipped with a high-efficiency particulate arrestance (HEPA) filter, or other HEPA-filtered masks (e.g., N95, if no facial hair is present) in conjunction with a facial splash guard can decrease likelihood of exposure to aerosolized particles and splashes that can transmit a variety of pathogens. Depending on the PAPR type, chemical filters can often be used when working with inhaled anesthetics. However, the purpose of this document is not to provide recommendations or guidelines regarding specific types of PPE, so we defer to institutional requirements and subject matter experts. In addition to the documents cited within this text, a variety of resources are listed in Appendix 1, which offer greater detail regarding selection of appropriate PPE and techniques for minimizing exposure risks.

The use of appropriate PPE has the potential to safeguard researchers from exposure to infectious agents, but it is not infallible (Fischer et al., 2014). Improper decontamination and doffing techniques can expose the wearer even after active handling has been completed. This suggests that proper training regarding donning and doffing protocols is as important, if not more so, than the presence of PPE itself. A proper discussion and walk-through consisting of multiple doffing techniques can be found at a number of online resources (Appendix 1). It is recommended that doffing procedures be standardized to assure that all participants will use recommended techniques consistently (Fischer et al., 2014).

Given the results of relatively recent surveys of professional organizations and National Park Service employees, it is clear that a large portion of wildlife biologists work consistently without gloves, HEPA masks, or disinfectants (Fulhorst et al., 2007; Kelt et al., 2007; Bosch et al., 2013), which are some of the least invasive and most effective pieces of PPE available; in addition, many wildlife biologists are not even aware of the need for PPE (Bosch et al., 2013). Reasons cited for the lack of PPE use include items being inconsistently stocked or available, invasive in extreme conditions (such as high heat and humidity), and costly (Bosch et al., 2013). Many of these potential barriers to the use of PPE can be addressed through a number of steps, including increased education regarding potential risks to field biologists, epidemiologists, veterinarians, and other occupations with increased contact with small mammals (e.g., staff at wildlife rehabilitation centers) and allocating a portion of course fees or grant monies toward PPE and proper training. Additionally, professional associations (e.g., The Wildlife Society, Wildlife Disease Association, American Society of Mammalogists, Latin American Mammal Congress) could serve as advocates for cultural change across professions (Bosch et al., 2013). Supervisor (principal investigator [PI]) engagement has been shown to increase compliance with work safety measures (Bosch et al., 2013; Lombardi et al., 2009). The first step toward reducing barriers that impede the use of PPE is to discuss the risks of various types of fieldwork and to consider ways to minimize those risks for all involved. We hope that as new professionals and students prepare to enter the fields of biological and epidemiological research, the topics of biosafety and risk mitigation are fresh on their minds. With knowledge in these areas, researchers in these important fields can continue to increase our understanding of mammalian biodiversity, zoonoses, and epidemiology while responsibly protecting themselves.

## Appendix I

A list of resources (in addition to those cited within the text) for more detailed information regarding biosafety in the field. These references include information from specific pathogens endemic to certain countries and prevention/ treatment measures, to donning/doffing procedures, and selection of appropriate PPE.

- Mills, J. N., Carroll, D. S., Revelez, M. A., Amman, B. R., Gage, K. L., Henry, S., & Regnery, R. L. (2007). Minimizing infectious disease risks in the field. Wildlife Professional, 1(4), 30.
- CDC Traveler’s Health page: contains information regarding endemic diseases, ongoing outbreaks, special prophylaxis recommendations for countries around the world: http://wwwnc.cdc.gov/travel/
- CDC Yellowbook: updated annually – can be purchased as a hard copy or electronic version. For more information visit website - http://wwwnc.cdc.gov/travel/page/yellowbook-home-2014
- WHO country information (http://www.who.int/countries/en/)
- Armed Forces Pest Management Board Technical Guide No. 41: Protection from rodent-borne diseases with emphasis on occupational exposure to hantavirus: contains information regarding a variety of pathogens, their reservoirs, geographic distributions, and routes of transmission, as well as risk mitigation for hantaviruses http://www.afpmb.org/sites/default/files/pubs/techguides/tg41.pdf
- CDC veterinary safety and health website: includes information on various zoonoses, links to other sites and documents for information on PPE, biological waste disposal: http://www.cdc.gov/niosh/topics/veterinary/biological.html#waste
- The online version of the Biosafety in Microbiological and Biomedical Laboratories (BMBL – 5^th^ edition) includes information regarding various agents for use in sterilization and disinfection of spaces and surfaces, as well as use of appropriate biosafety in laboratory conditions. http://www.cdc.gov/biosafety/publications/bmbl5/
- An example of an Institutional Animal Care and Use Committee Standard Operating Procedure from the University of Colorado, Boulder website: http://www.colorado.edu/vcr/sites/default/files/attached-files/SOP%2015%20PPE%20Policy%2008072013_0.pdf
- The National Park Service’s page for Vectorborne & Zoonotic Infectious Agents includes links for information on various pathogens, their vectors, potential hosts, and safe practices to avoid zoonotic disease from wildlife: https://www.nps.gov/public_health/di/vb_ia.htm
- World Health Organization’s Rapid advice guidelines: Personal protective equipment in the context of filovirus disease outbreak response. http://apps.who.int/iris/bitstream/10665/137410/1/WHO_EVD_Guidance_PPE_14.1_eng.pdf?ua=1
